# Reproducing the dopamine pathophysiology of schizophrenia and approaches to ameliorate it: a translational imaging study with ketamine

**DOI:** 10.1038/s41380-020-0740-6

**Published:** 2020-05-07

**Authors:** Michelle Kokkinou, Elaine E. Irvine, David R. Bonsall, Sridhar Natesan, Lisa A. Wells, Mark Smith, Justyna Glegola, Eleanor J. Paul, Kyoko Tossell, Mattia Veronese, Sanjay Khadayate, Nina Dedic, Seth C. Hopkins, Mark A. Ungless, Dominic J. Withers, Oliver D. Howes

**Affiliations:** 1 MRC London Institute of Medical Sciences (LMS), London, W12 0NN UK; 2 Institute of Clinical Sciences (ICS), Faculty of Medicine, Imperial College London, London, W12 0NN UK; 3 Invicro, Burlington Danes, Hammersmith Hospital, London, W12 0NN UK; 4 Department of Psychosis Studies, Institute of Psychiatry, Psychology and Neuroscience, Kings College London, London, UK; 5 Department of Neuroimaging, Institute of Psychiatry, Psychology and Neuroscience, Kings College London, London, UK; 6 Sunovion Pharmaceuticals, 84 Waterford Drive, Marlborough, MA 01752 USA

**Keywords:** Schizophrenia, Neuroscience

## Abstract

Patients with schizophrenia show increased striatal dopamine synthesis capacity in imaging studies. The mechanism underlying this is unclear but may be due to N-methyl-D-aspartate receptor (NMDAR) hypofunction and parvalbumin (PV) neuronal dysfunction leading to disinhibition of mesostriatal dopamine neurons. Here, we develop a translational mouse model of the dopamine pathophysiology seen in schizophrenia and test approaches to reverse the dopamine changes. Mice were treated with sub-chronic ketamine (30 mg/kg) or saline and then received in vivo positron emission tomography of striatal dopamine synthesis capacity, analogous to measures used in patients. Locomotor activity was measured using the open-field test. In vivo cell-type-specific chemogenetic approaches and pharmacological interventions were used to manipulate neuronal excitability. Immunohistochemistry and RNA sequencing were used to investigate molecular mechanisms. Sub-chronic ketamine increased striatal dopamine synthesis capacity (Cohen’s *d* = 2.5*)* and locomotor activity. These effects were countered by inhibition of midbrain dopamine neurons, and by activation of PV interneurons in pre-limbic cortex and ventral subiculum of the hippocampus. Sub-chronic ketamine reduced PV expression in these cortical and hippocampal regions. Pharmacological intervention with SEP-363856, a novel psychotropic agent with agonism at trace amine receptor 1 (TAAR1) and 5-HT_1A_ receptors but no appreciable action at dopamine D_2_ receptors, significantly reduced the ketamine-induced increase in dopamine synthesis capacity. These results show that sub-chronic ketamine treatment in mice mimics the dopaminergic alterations in patients with psychosis, that this requires activation of midbrain dopamine neurons, and can be ameliorated by activating PV interneurons and by a TAAR1/5-HT_1A_ agonist. This identifies novel therapeutic approaches for targeting presynaptic dopamine dysfunction in patients with schizophrenia and effects of ketamine relevant to its therapeutic use for  treating major depression.

## Introduction

Schizophrenia is a severe mental disorder and a significant global health burden, highlighting the need to better understand its neurobiology in order to develop improved treatments [1]. Dopaminergic hyperactivity in the striatum is thought to underlie the symptoms of schizophrenia, particularly psychosis [2–5]. Supporting this, 3,4-dihydroxy-6-^18^F-fluoro-l-phenylalanine ([^18^F]-FDOPA) positron emission tomography (PET) imaging studies have revealed higher striatal dopamine synthesis capacity in patients with schizophrenia [6–9]. Furthermore, increased dopamine synthesis capacity is associated with both the development of psychosis [10] and the severity of symptoms [11]. Currently available antipsychotics are all dopamine receptor blockers, which are inadequate and poorly tolerated in many patients, and do not address the mechanism underlying the dopamine dysfunction [12, 13]. In addition to dopaminergic dysfunction, the glutamate hypothesis of schizophrenia has developed from the observations that N-methyl-D-aspartate receptor (NMDAR) antagonists such as ketamine induce psychotic symptoms in healthy humans and exacerbate symptoms in patients [14, 15]. Furthermore, schizophrenia is associated with a reduction in parvalbumin (PV)-expressing GABAergic interneurons, which are regulated by NMDARs in the cortex and hippocampus [16–18]. It has been suggested that impaired PV neuronal function in the cortex and hippocampus may lead to disinhibition of mesostriatal dopamine neuron activity via a polysynaptic pathway [19]. However, it is not clear if it is possible to develop a pre-clinical model of the increased dopamine synthesis capacity seen in patients using an NMDAR antagonist, and whether it is possible to reverse this by targeting PV-expressing interneurons or other mechanisms.

To address this, we tested the effect of sub-chronic ketamine administration on dopamine synthesis capacity in mice using the same [^18^F]-FDOPA PET imaging technique that previously demonstrated elevation in dopamine synthesis capacity in patients [6–9], and also tested the potential of activating PV-interneurons to reverse the effects of ketamine on striatal dopamine synthesis capacity. We also tested the translational potential of a novel psychotropic agent, SEP-0363856 (SEP-856), to reverse striatal dopaminergic alterations based on evidence that it inhibits ventral tegmental area (VTA) neuronal firing [20]. Our objective was to develop a chemogenetics/PET approach that is translationally relevant and provides novel insights into the pathophysiology of schizophrenia.

## Methods and materials

All experiments were approved by the UK Home Office under the Animal (Scientific Procedures) Act (ASPA) 1986 and Regulation 7 of the Genetically Modified Organisms (Contained Use) Regulations 2000. All procedures were performed in accordance with the ASPA 1986 and EU directive 2010/63/EU as well as being approved by Imperial College Animal Welfare and Ethical Review Body.

### Subjects

Male mice were 6–8 weeks of age at the time of stereotaxic surgeries and 8–10 weeks of age at the start of the experiments. C57BL/6 wild-type, dopamine transporter (DAT) Cre *(DAT::Cre)* and parvalbumin (PV) Cre *(PV::Cre)* mice maintained on a C57BL/6 background were used.

### Sub-chronic ketamine regime

**Fig. 1:**
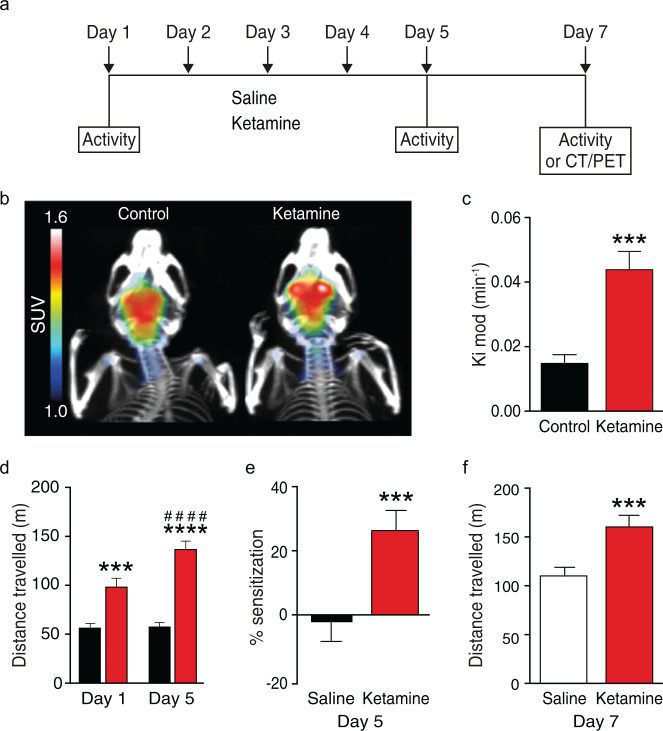
Sub-chronic ketamine increases dopamine synthesis capacity and locomotor activity. **a** Schematic showing the drug treatment schedule used to study the effect of sub-chronic ketamine administration on striatal dopamine synthesis capacity and locomotor activity in the mouse. **b** [^18^F]-FDOPA PET brain image coregistered to a computed tomography (CT) scan demonstrating high signal to noise ratio in striatal uptake from representative mice treated with saline or ketamine, and showing increased uptake in ketamine-treated mice. Standardised uptake value (SUV) is presented as summed activity over the timeframe (20–90 min) used to measure dopamine synthesis capacity. **c** Striatal dopamine synthesis capacity (*K*_i_^*mod*^/min) is significantly increased in the ketamine-treated (*n* = *8*) group versus control (*n* = *7*) group (***P < 0.001, two-tailed, Cohen’s *d* = 2.5, *t*_13_ = 4.74). **d** Total distance travelled during 30 min post drug administration. There was a significant effect of group (*F*_(1, 26)_ = 46.21, *P* < 0.0001), day (*F*_(1, 26)_ = 23.27, *P* < 0.0001) and group × time interaction (*F*_(1, 26)_ = 20.79, *P* < 0.001; Bonferroni post hoc (asterisks indicate *p*-values for saline vs ketamine on the same day; hash indicates *p*-values for day 1 vs day 5), showing that ketamine induces hyperlocomotion. **e** Sub-chronic ketamine induces locomotor sensitisation (***P < 0.001). **f** Locomotor sensitization is sustained following a 2-day washout of ketamine. Ketamine induced significantly higher locomotor activity in mice that had received sub-chronic ketamine as compared with mice that had received saline for 5 days (***P < 0.001). Data represent mean ± S.E.M. ****P < 0.0001; *** *P* < 0.001; #### *P* < 0.0001. PET positron emission tomography, CT computed tomography.

Ketamine hydrochloride solid (Sigma-Aldrich) was dissolved in 0.9% saline solution to 6 mg/ml and injected at a volume of 5 ml/kg of body weight, thus administered at a dose of 30 mg/kg (i.p) once daily for 5 consecutive days (Fig. 1a, Supplementary Figs. 1a, 2a, 3a, 4) [21]. Control mice received an equivalent volume of 0.9% saline vehicle.

### Chemogenetics model

**Fig. 2:**
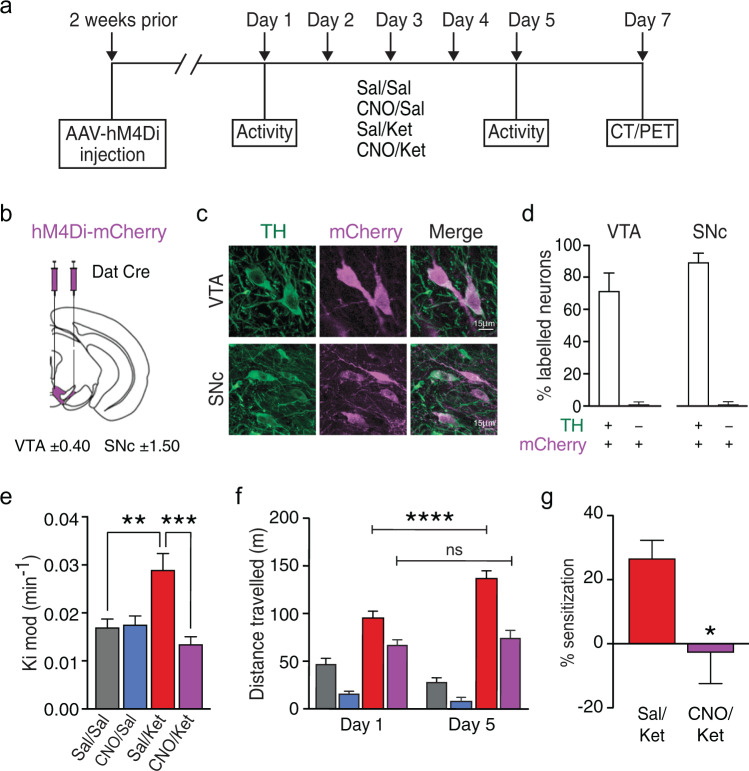
Midbrain dopamine neuron firing is necessary for ketamine-induced increases in dopamine synthesis capacity and locomotor activity. **a** Experimental timeline and drug treatment paradigm used to assess the effect of midbrain dopamine neuron inhibition on the sub-chronic ketamine-induced increase in striatal dopamine synthesis capacity and locomotor activity. Two weeks after stereotaxic injection of AAV-hM4Di-mCherry, mice received 0.1 mg/kg CNO or vehicle followed by ketamine (30 mg/kg) or vehicle 30 min later for 5 consecutive days. Mice underwent a dynamic PET/CT scan 2 days after the last drug administration. **b** Bilateral infusion of AAV-hM4Di-mCherry into the VTA and SNc of *DAT::Cre* mice was used to selectively express DREADD receptors in dopamine neurons. **c** Fluorescence confocal images of representative midbrain fields depicting co-expression (white) of mCherry (magenta) and TH (green)  immunofluorescence. **d** Percentage of TH+ neurons co-expressing mCherry (47 out of total 65 TH+ neurons; 71.7 ± 11%) and percentage of mCherry+ which do not express TH (1 out of total 48 mCherry + neurons, 1.5 ± 1.5%) in the VTA. Percentage of TH+ neurons co-expressing mCherry (47 out of total 52 TH+ neurons; 89.6% ± 5.4) and percentage of mCherry+ which do not express TH (1 out of total 48 mCherry+ neurons, 1.5 ± 1.5%) in the SNc. **e** Striatal dopamine synthesis capacity (*K*_i_^*mod*^/min) is significantly reduced in CNO/Ket compared with Sal/Ket group (***P < 0.001) (Sal/Sal (*n* = 12), CNO/Sal (*n* = 13), Sal*/*Ket (*n* = 12) and CNO/Ket-treated (*n* = 11) groups). **f** Total distance travelled during 30 min post drug administration. Sub-chronic ketamine treatment induced locomotor sensitization that was prevented by inhibition of midbrain dopamine neuron firing prior to ketamine treatment. **g** Percentage locomotor sensitization between day 1 and day 5. Data represent mean ± S.E.M. ****P < 0.0001; ***P < 0.001; **P < 0.01; *P < 0.05. Sal saline, Ket ketamine, CNO clozapine N-oxide, TH tyrosine hydroxylase, PET positron emission tomography, CT computed tomography, VTA ventral tegmental area, SNc substantia nigra pas compacta.

**Fig. 3:**
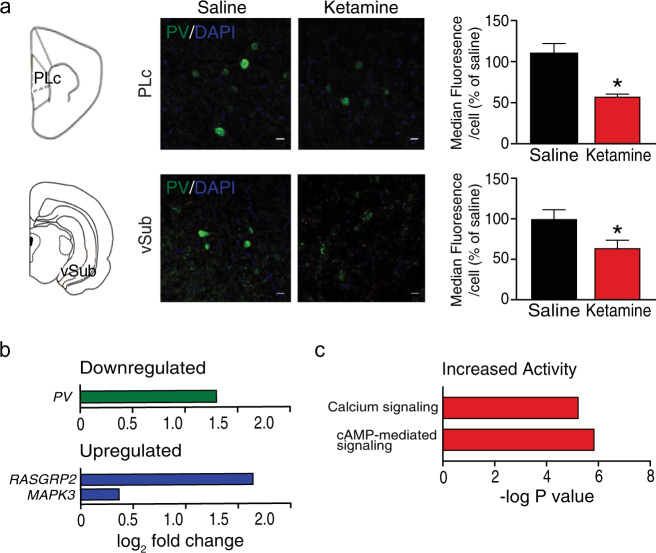
Sub-chronic ketamine reduces parvalbumin interneuron function. **a** Schematics of the location of the PLc and vSub of the hippocampus in the brain. Representative fluorescence confocal images of  PLc and vSub of the hippocampus fields respectively depicting PV interneuron (green) and DAPI (blue) immunofluorescence in saline-treated (Saline) and ketamine-treated (Ketamine) mice. PV immunofluorescence in the PLc and vSub of the hippocampus is significantly reduced in the ketamine versus saline group (Two-way repeated-measures ANOVA significant effect of treatment, *F*_1, 8_ = 47.28, *p* < 0.001, *η*^2^ effect size = 0.86; followed by Bonferroni post hoc tests (*P* < 0.05); *n* = 5 mice per group). **b** Differential expression of the PV gene in ketamine-treated mice vs. saline treated controls. Differential expression of RASGRP2 and MAPK3 genes in ketamine-treated mice vs. saline treated controls. Log2 fold change is shown in each respective bar. **c** Increased activity in the calcium signalling and cAMP-mediated signalling pathways in ketamine-treated vs. control group. Data represent mean ± S.E.M. *P < 0.05. PLc pre-limbic cortex, vSub ventral subiculum of the hippocampus, PV parvalbumin.

**Fig. 4:**
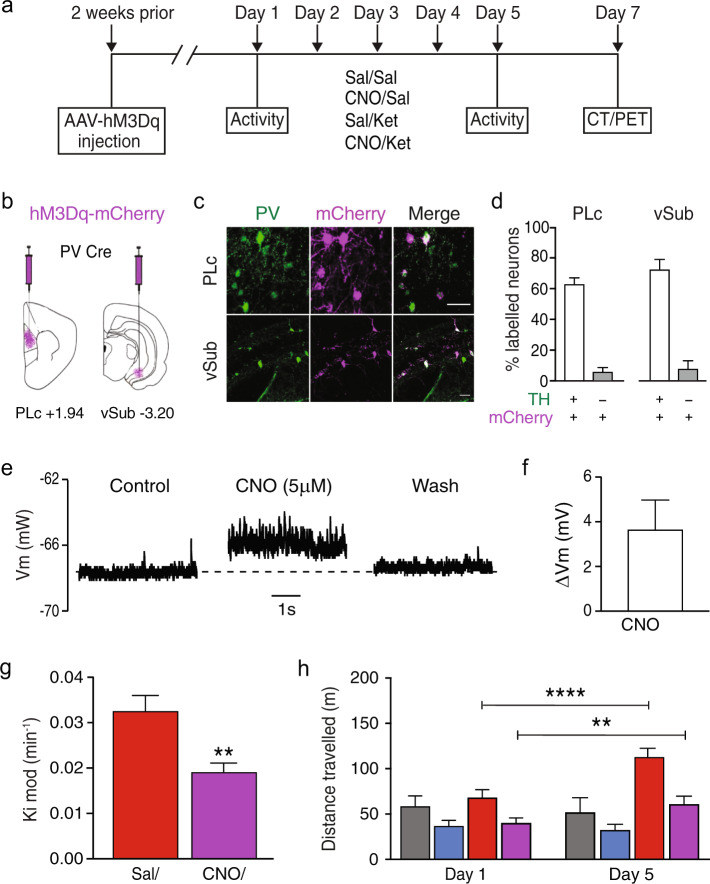
In vivo parvalbumin interneuron activation attenuates the effects of sub-chronic ketamine-induced increase in dopamine synthesis capacity and locomotor activity. **a** Experimental timeline and drug treatment schedule used to study the effect of PV neuron activation on the sub-chronic ketamine-induced increase in striatal presynaptic dopamine synthesis capacity. Two weeks following stereotaxic injection of AAV-hM3Dq-mCherry, mice received 0.5 mg/kg CNO or vehicle, followed by ketamine (30 mg/kg) or vehicle treatment 30 min later for 5 consecutive days. Mice underwent a dynamic PET/CT scan 2 days following the last drug administration. **b** Bilateral infusion of AAV-hM3Dq-mCherry into the PLc and vSub of *PV::Cre* mice was used to selectively express DREADD receptors in PV interneurons. **c** Representative fluorescence confocal images of PLc and vSub fields depicting co-expression (white) of mCherry (magenta) and PV (green) immunofluorescence. **d** Percentage of PV+ neurons co-expressing mCherry (120 out of total 177 PV+ neurons; 65 ± 4.4%) and percentage of mCherry+ which do not express PV (9 out of total 129 mCherry+ neurons, 5.8 ± 2.9%) in the PLc. Percentage of PV+ neurons co-expressing mCherry (51 out of total 71 PV+ neurons; 72.9 ± 6.3%) and percentage of mCherry+ which do not express PV (5 out of total 59 mCherry+ neurons, 7.8 ± 5.2%) in the vSub. **e** Effect of 5 microM CNO on membrane potential measured in voltage clamp configuration from a whole-cell recording of PV interneuron within the vSub from a *PV::Cre* mouse injected with AAV-hM3Dq-mCherry. **f** Change in membrane potential with positive showing increase relative to the baseline indicative of PV neuron depolarisation upon CNO application. **g** Striatal dopamine synthesis capacity (*K*_i_^*mod*^/min) is significantly reduced in CNO/ketamine-treated (*n* = 11) (purple) versus SAL/Ket (*n* = 11) (red) group, unpaired t-test (**P < 0.01, *t*_19_ = 3.51, two-tailed, effect size = 1.59). **h** Total distance travelled during a 30 min test period post drug administration. Sub-chronic ketamine treatment induced locomotor sensitization, which was not prevented by activation of PV interneuron firing prior to ketamine treatment (*F*_1, 44_ = 15.51, *** *P* < 0.001, Bonferroni post hoc test **P < 0.01 ***P < 0.001 = day 1 vs. day 5). On day 5 activation of PV interneuron firing prevented the effects of sub-chronic ketamine on locomotor activity (*F*_3, 44_ = 9.283, ***P < 0.001, Bonferroni post hoc test ***P < 0.001 Sal/Ket vs. all other groups). Data represent mean ± S.E.M. ****P < 0.001; **P < 0.01. Sal saline, Ket ketamine, CNO clozapine N-oxide, PET positron emission tomography, CT computed tomography, PLc pre-limbic cortex, vSub ventral subiculum of the hippocampus, PV parvalbumin.

In *DAT::Cre* mice adeno-associated virus (AAV) vectors were stereortaxically targeted to the ventral tegmental area (VTA: anteroposterior [AP] −3.15 mm, mediolateral [ML] ±0.40 mm, dorsoventral [DV] −4.30 mm) and the substantia nigra pars compacta (SNc: AP −3.15 mm, ML ±1.50 mm, DV −4.30 mm) (Fig. 2b). In *PV::Cre* mice viruses were stereotaxically injected in pre-limbic cortex (PLc: AP +1.94, ML ±0.45, DV −2.20) and in the ventral subiculum (vSub) of the hippocampus (vSub: AP −3.20, ML ±2.80, DV −4.30) (Figs. 3a, 4b, Supplementary Fig. 5). Both the PLc and vSub regions were targeted concurrently for the following reasons. In schizophrenia there are deficits in PV interneuron markers in both the prefrontal cortex and in the hippocampus [16, 22–25]. In addition, acute and chronic ketamine administration is associated with deficits in PV interneuron markers in both regions [16, 17, 26–29]. The needle was left in place for 3 min post injection. Following injections, the wound was sutured (Mersilk, 3–1 Ethicon). Two weeks following the surgeries, clozapine N-oxide (CNO) (0.1 and 0.5 mg/kg, i.p) or saline was administered 30 min before the injection of ketamine or saline (Figs. 2a, 4a). See Supplementary Methods for further details.

### Open-field test

Mice were placed into the open-field arena for a 20 min habituation period, then injected i.p with either ketamine or saline and placed back in the arena for a further 60 min. Total distance travelled was recorded using the Ethovision XT video tracking software system (Noldus Information Technologies, Leesburg, VA, USA). Locomotor activity was assessed on days 1, 5 and 7 of drug treatment (Supplementary Fig. 1). For the chemogenetic experiments, mice were placed in the open-field arenas for 20 min habituation, they then received an injection of CNO or saline and activity was recorded for 30 min. Then, mice received an injection of ketamine or saline, in line with the treatment schedule, and their activity recorded for 60 min. Locomotor activity was assessed on days 1 and 5 of drug treatment (Supplementary Figs. 2–4).

### Positron emission tomography (PET) imaging

One hour prior to scanning, mice were anaesthetised with isoflurane and underwent external jugular vein cannulation. During scanning, the respiration rate was monitored using the BioVet physiological monitoring software system (Biovet software; m2m Imaging Corp, Cleveland, OH, USA) and body temperature was maintained at 37 °C. Mice received 40 mg/kg (i.p) entacapone (SML0654, Sigma-Aldrich), a catechol-O-methyl-transferase inhibitor, and 10 mg/kg (i.p) benserazide hydrochloride (B7283, Sigma-Aldrich), an aromatic amino acid decarboxylase inhibitor, at 45 and 30 min before the [^18^F]-FDOPA respectively. This improves brain uptake [^18^F]-FDOPA by reducing peripheral metabolism of the radiotracer [30]. SEP-363856 (3 mg/kg, i.p), a TAAR1/5HT_1A_ *agonist*, was provided by Sunovion Pharmaceuticals and administered 30 min prior to the [^18^F]-FDOPA injection. Following cannulation, mice were transferred to the bore of an Inveon µPET/CT scanner (Siemens, Surrey, UK). Mice underwent a 20 min CT scan for attenuation correction, and then received a bolus injection of ~4.5 MBq [^18^F]-FDOPA via the external jugular vein cannula at the start of the 120 min dynamic PET scan.

### PET analysis

Inveon Research Workplace software (Siemens USA) was used to draw 3D regions of interest manually on summation radioactivity images at the level of the striatum (right and left) (0.07 cm^3^) and the cerebellum (0.1 cm^3^) to extract time activity curves (Supplementary Figs. 6, 7) [31]. Dopamine synthesis capacity was indexed as the rate constant for the uptake and conversion of [^18^F]-FDOPA to [^18^F]-dopamine, *K*_i_^*mod*^ (min^−1^), and determined using a modified Patlak plot accounting for the loss of radioactive metabolites, *k*_loss_ [30, 32]. The cerebellum was used as the reference region, in line with the approach used in human studies, to account for non-specific uptake as it has negligible dopaminergic projections [33, 34].

### RNA sequencing (RNA Seq)

Two days following 5 days of ketamine or saline injections, brains were rapidly removed and the PLc was dissected and frozen in isopentane on dry ice. Total RNA was isolated using the TriZol reagent (Invitrogen) and purified using RNAeasy micro kits from Qiagen. RNA integrity was assessed using the Agilent Bioanalyser and all RNA integrity number values were above 8. Then, the cDNA library was prepared using the NEB Next Ultra II Library Prep kit (New England Biolabs, USA). Sequencing was conducted on an Illumina HiSeq 2500 system with 100 base pair paired-end reads (London, UK). Raw reads were aligned to mm9 genome using Tophat version (2.0.11) [35]. Gene based counting was performed using the HTSeq counts module. Gene expression analysis was performed using the DESeq2 Bioconductor package. All genes with adjusted *p* value of 0.05 or less (calculated from the raw *p* values using the Benjamini and Hochberg algorithm) were considered statistically significant. The RNA seq data are available at https://www.ncbi.nlm.nih.gov/geo/query/acc.cgi?acc=GSE138802.

### Statistical analysis

Statistical analyses were performed using Prism 7.00 software (GraphPad Software, La Jolla, California, USA). Normality of distributions was assessed using the Kolmogorov–Smirnov test and Levene’s test for equality of variance to guide the choice of statistic. Between-group comparisons were made with two-tailed independent samples *t* tests for normally distributed data, and Mann–Whitney *U* tests were used for non-parametric data. Two-way analysis of variance (ANOVA) was used to test the difference in outcome measure between the four experimental groups. Locomotor sensitization was analysed with a two-way repeated-measures ANOVA, with the days as the repeated measure and experimental group as the cofactor. Outliers in the data were identified using the Grubbs’s test. Post hoc comparisons were Bonferroni corrected. Cohen’s *d* effect sizes were calculated using the online calculator (http://www.uccs.edu/~lbecker/). Data are expressed as mean ± s.e.m. and statistical significance was defined as *p* < 0.05 (two tailed).

## Results

### Sub-chronic ketamine increases dopamine synthesis capacity and locomotor activity

To test the hypothesis that sub-chronic ketamine administration leads to increased dopamine synthesis capacity, mice were injected once daily with ketamine (30 mg/kg) or saline for 5 consecutive days. Two days after the last ketamine or saline injection in vivo [^18^F]-FDOPA PET imaging was performed. Sub-chronic ketamine treatment significantly increased striatal dopamine synthesis capacity compared to controls, with an effect size of *d* = 2.5 (*P* < 0.001, *t*_13_ = 4.74) (Fig. 1b, c, Supplementary Table 1). We also examined locomotor sensitization, which has been used as a behavioural model of the dopaminergic dysfunction seen in psychosis [36]. Acute ketamine administration (day 1) induced locomotor hyperactivity in the open-field test. Repeated ketamine administration (day 5) induced locomotor sensitization, an effect that was sustained following 2-day washout of ketamine (day 7) (Fig. 1d–f; Supplementary Fig. 1). Collectively, these findings indicate that sub-chronic ketamine administration induces both an increase in dopamine synthesis capacity and behavioural changes relevant to schizophrenia.

### Midbrain dopamine neuron firing is necessary for ketamine-induced increases in dopamine synthesis capacity and locomotor activity

To test if the reported ketamine-induced firing activity of dopamine neurons [37–39] underlies the increase in dopamine synthesis capacity we observed, we employed a chemogenetic approach to selectively suppress dopamine neuron activity in vivo. We injected an adeno-associated virus (AAV) containing a Cre-dependent *hM4Di-mCherry* fusion protein (AAV1-DIO-hM4Di-mCherry) into the VTA and the substantia nigra pars compacta (SNc) of *DAT::Cre* mice. Cre-dependent expression of *hM4Di-mCherry* showed ~98% specificity for dopamine neurons, and CNO-treatment silenced dopamine neuron firing in slice electrophysiology recordings, consistent with our previous findings (Fig. 2b–d) [40]. Administration of CNO prior to ketamine dosing prevented the elevation in striatal dopamine synthesis capacity (Fig. 2e, Supplementary Table 2) and the ketamine-induced locomotor sensitization compared to the relevant control groups (Fig. 2f, g, Supplementary Fig. 2). It has recently been shown that clozapine, converted from CNO, may have off-target effects at endogenous receptors rather than at the DREADDs exclusively [41]. Importantly, CNO administration in transgenic mice expressing a control construct had no effect on the ketamine-induced increase in dopamine synthesis capacity and locomotor activity (Supplementary Fig. 4), indicating that DREADD-mediated silencing of dopaminergic neurons is responsible for the observed effects. Taken together, these findings suggest that sub-chronic ketamine increases dopamine synthesis capacity and locomotor sensitization through a mechanism that drives firing activity of midbrain dopamine neurons.

### The effect of sub-chronic ketamine on PV expression and function

Lower levels of PV neurons in the cortex and hippocampus have been observed in schizophrenia patients and following acute ketamine treatment [16, 17, 27, 42]. In addition, it is believed that reduced PV neuron function may lead to changes in dopamine neuron activity [19]. Therefore, we examined the effects of ketamine on various elements of PV interneuron function including PV expression. We found that sub-chronic ketamine treatment reduced PV interneuron immunofluorescence in the pre-limbic cortex (PLc) and the ventral subiculum (vSub) of the hippocampus (*P* < 0.05, *η*^2^ effect size = 0.86) relative to saline controls (Fig. 3a).

To investigate the molecular mechanisms underlying the effects of sub-chronic ketamine on dopamine synthesis we performed RNA Seq on PLc tissue. We hypothesised that sub-chronic ketamine would result in reduced PV expression, and changes in signalling pathways downstream of the NMDA receptor such as calcium signalling and the activation of brain-derived neurotrophic factor (BDNF) signalling. Consistent with our a priori hypotheses, RNA Seq data on PLc tissue revealed reductions in the expression of PV (Fig. 3b). Moreover, consistent with the hypothesis that blocking NMDAR activity increases BDNF signalling [43], we observed a significant increase in the expression of genes involved in the pathway downstream of BDNF signalling, specifically upregulation of mitogen-activated protein kinase 3 (MAPK3) and RAS guanyl releasing protein 2 (Rasgrp2) (Fig. 3b). In addition, cAMP-mediated signalling and calcium signalling pathways were significantly activated in ketamine vs. saline conditions (Fig. 3c). Furthermore, using Ingenuity-pathway analysis (IPA, QUIAGEN Redwood City, https://www.qiagenbioinformatics.com/products/ingenuity-pathway-analysis/) L-DOPA was the significant upstream regulator of the differentially expressed genes in ketamine vs. saline conditions (*z*-score = 2.961, *P* < 0.05, in cortex). Collectively, these data support the hypothesis that sub-chronic ketamine increases dopamine synthesis capacity via a pathway that involves the inhibition of PV interneuron function. Furthermore, RNA Seq data on PLc tissue did not reveal changes in the expression of cholecystokinin (*p* > 0.05), nitric oxide synthase 1 (*p* > 0.05) and somatostatin (*p* > 0.05), which are proteins expressed in other GABAergic interneurons. Interestingly, consistent with a previous study, vasoactive intestinal peptide receptor 2 (VIPR2) expression was significantly increased suggesting that other GABAergic interneurons may be affected by sub-chronic ketamine administration.

### The role of PV interneuron activity in mediating the effects of sub-chronic ketamine

Given that ketamine reduced PV expression levels, and the hypothesised role of PV interneuron hypofunction in schizophrenia [44, 45], we aimed to determine if activating PV interneurons in the PLc and vSub of the hippocampus was able to counter the ketamine-induced increase in dopamine synthesis capacity. To test this, AAVs expressing a Cre-dependent *hM3Dq-mCherry* fusion protein were stereotaxically injected into the PLc and vSub of the hippocampus of *PV::Cre* mice (Fig. 4a, b). Immunohistochemistry revealed co-localisation of mCherry with PV immunoreactive neurons and a successful transduction with over 92% specificity in the PLc and vSub (Fig. 4c, d). In ex vivo slice electrophysiology studies, application of CNO depolarised vSub PV neurons expressing mCherry (Fig. 4e, f). Using this system, we found that in vivo activation of PV interneurons in the PLc and vSub, prior to ketamine administration, significantly reduced both the elevation in striatal dopamine synthesis capacity (*P* < 0.01, *t*_19_ = 3.51, two-tailed, effect size = 1.59) (Fig. 4g; Supplementary Table 3) and the locomotor effects of acute and sub-chronic ketamine (Fig. 4h; Supplementary Fig. 3). Therefore, our results suggest that ketamine increases dopamine synthesis capacity and locomotor activity via its actions on cortical/hippocampal PV interneurons.

### A novel TAAR1/5-HT_1A_ agonist counteracts the ketamine-induced increase in dopamine synthesis capacity

**Fig. 5:**
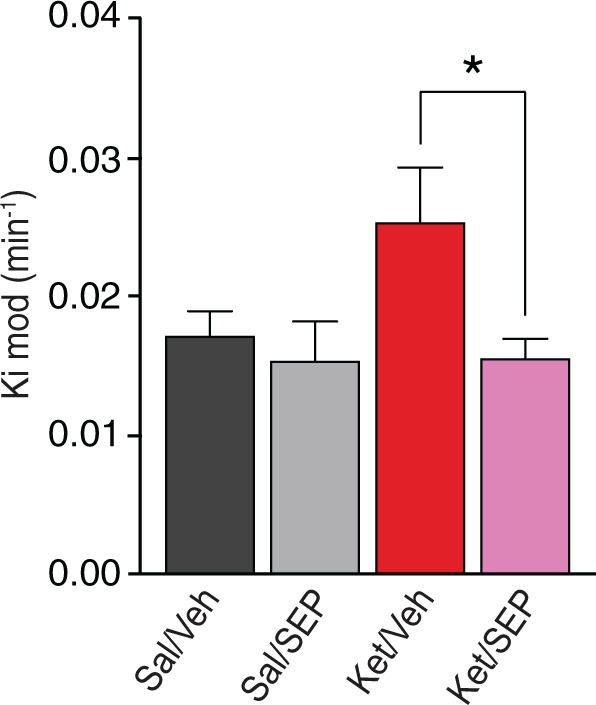
The novel trace amine receptor 1 agonist SEP-0363856 (SEP-856) attenuates the ketamine-induced increase in dopamine synthesis capacity. Striatal dopamine synthesis capacity (*K*_i_^*mod*^/min) was significantly reduced in the ketamine model following the administration of SEP compared with vehicle in the ketamine model. Ket/SEP (*n* = 9) (Magenta) versus Ket/Veh (*n* = 8) (red) *P < 0.05. Sal saline, Ket ketamine, Veh vehicle, SEP SEP-0363856.

Our findings suggest that targeting dopamine neuron firing activity may present a viable therapeutic target for the increase in dopamine synthesis capacity seen in schizophrenia. One potential candidate mechanism is trace amine receptor 1 (TAAR1) agonism. TAAR1 is a G-protein-coupled receptor that is expressed throughout monoaminergic brain nuclei including dopamine neurons [46]. TAAR1 agonists have been shown to reduce dopamine firing rates and release [47–49]. In view of this, we tested whether SEP-0363856 (SEP-856), a novel psychotropic agent with agonism at TAAR1 and  5HT_1A  _ receptors [20], counteracts the effect of sub-chronic ketamine treatment on dopamine synthesis capacity. Ketamine-treated mice that received SEP-856 (3 mg/kg, i.p) showed significantly lower striatal dopamine synthesis capacity compared to vehicle controls  (*P < *0.05, *t*_29_ = 2.839) (Fig. 5). Post hoc analyses showed that $K_{\mathrm{i}}^{mod}$ in ketamine-treated mice that received SEP-856 was not significantly different from $K_{\mathrm{i}}^{mod}$ in mice not exposed to ketamine (Fig. 5).

## Discussion

Our results demonstrate that sub-chronic ketamine administration leads to elevated striatal dopamine synthesis capacity, and that this requires the activation of midbrain dopamine neurons. The ketamine-induced increase in dopamine synthesis capacity was attenuated by the activation of PV interneurons in the PLc and vSub, as well as by a novel TAAR1/5HT_1A_ agonist, SEP-856. Our study demonstrates for the first time to our knowledge that an experimental model induces the same dopaminergic phenotype seen in patients with psychosis, and the potential of targeting PV interneurons and a novel TAAR1/5HT_1A_ agonist to reverse the dopaminergic phenotype.

The majority of studies show that acute ketamine significantly increases striatal dopamine levels [50–53], and elevates VTA dopamine neuron firing [38, 39, 54]. Our results extend these findings to show that sub-chronic ketamine induces a persistent elevation of dopamine synthesis capacity through a mechanism that requires midbrain dopamine neuron firing. These results are in line with the increased striatal dopamine synthesis capacity observed in schizophrenia patients and were acquired using an equivalent PET imaging technique. It should be noted that we investigated dopamine synthesis capacity in the striatum and, although it includes nucleus accumbens, most of the signal is from dorsal regions (caudate and putamen) [34]. Therefore, inferences about the dopamine system elsewhere (e.g. in cortical regions) cannot be made. In addition, we extend prior findings of reductions in PV levels in the hippocampus and prefrontal cortex following acute or sub-chronic ketamine administration [27, 42], to show that ketamine also leads to persistent reductions in PV levels and that activation of PV interneurons can attenuate ketamine-induced increases in striatal dopamine synthesis capacity.

### Proposed mechanism of action of ketamine on dopamine synthesis capacity

Ketamine is a non-competitive NMDAR antagonist that binds with high affinity (Ki = 3.1 µM) to the same binding site as MK801 and PCP [55–57]. PV interneurons are activated upon glutamate binding to NMDAR, and subsequently inhibit the activity of cortical pyramidal neurons [58–60]. Therefore, by blocking NMDARs, ketamine is thought to reduce the activity of PV interneurons and thereby disinhibit cortical pyramidal neurons, including neurons that project to subcortical regions to ultimately disinhibit midbrain dopamine neuron firing [19, 38, 61–63]. In line with this model of the mechanism of action of ketamine on the dopaminergic system, ketamine has been associated with a reduction in PV interneuron function, excessive glutamate release [64–67] and an increase in dopamine neuron firing [38, 39, 54]. In addition, lower GABAergic neural activity leads to a reduction in PV expression [68, 69] and lower PV expression has been correlated with a reduction in coordinated neuronal activity during task performance in rodents [70]. Specifically, PV may modulate GABA transmitter release by acting as an antifacilitation factor [71], where at lower PV concentrations, PV is acting as a buffer and at higher concentrations the free form of PV may become functionally relevant to have an effect on synaptic dynamics [72–74]. Our findings that ketamine’s effects can be reduced by activating PV interneurons and inhibiting midbrain dopamine neurons is consistent with this model. However, it remains possible that ketamine has actions on other neuronal populations that contribute to its effects on striatal dopamine synthesis capacity. Given the evidence of lower PV levels in the frontal cortex and hippocampus in schizophrenia [22–25] and that acute and chronic ketamine administration leads to lower PV levels in the frontal cortex and hippocampus [16, 17, 26–29], we targeted both regions in our chemogenetics experiment. However, a limitation of targeting both regions is that we are not able to distinguish the relative contribution of each region to the effects we observe. Future work targeting each region separately would be useful to determine this. In addition, we investigated the effect of ketamine on PV-positive GABAergic interneurons because this subtype has been specifically implicated in schizophrenia pathophysiology [16, 17]. Notwithstanding this, alterations in other interneurons, particularly somatostatin-positive interneurons, have also been associated with schizophrenia [75]. However, we did not see expression changes in cholecystokinin (*p* > 0.05), nitric oxide synthase 1 (*p* > 0.05) or somatostatin (*p* > 0.05) in our RNA seq data, suggesting that ketamine does not have major effects on these interneurons in the PLc, although we cannot exclude effects in other brain regions. In contrast, our RNA seq data revealed a significant increase in the expression of VIPR2 in the PLc. This extends a previous finding showing this following acute ketamine administration [76], to indicate that VIPR2 expression is also increased after sub-chronic ketamine administration. VIPR2 is expressed in somatostatin-positive interneurons and increases excitability of these interneurons [76]. This finding highlights that other GABAergic interneurons may be affected by sub-chronic ketamine treatment and the need for further work to determine if expression changes in these interneurons, or others that we were not able to measure such as calretinin positive interneurons, contribute to ketamine’s effects on dopamine regulation and behaviour.

Moreover, there is evidence of direct glutamatergic projections from the PLc and other frontal regions to the substantia nigra/VTA that activate dopamine neurons and increase locomotor behaviour in an NMDAR dependent manner [77, 78]. The PLc also projects to the lateral habenula [79, 80], which is another major source of glutamatergic projections to the rostromedial tegmental nucleus [81]. In addition, both the PLc and the vSub activate neurons in the amygdala and related regions including the bed nucleus of stria terminalis, which project to and may activate VTA dopamine neurons [82, 83]. Glutamatergic projections from the vSub to the nucleus accumbens also activate midbrain dopamine neurons in an NMDAR dependent manner through a pathway involving the ventral pallidum [84]. Furthermore, glutamatergic projections from the pedunculopontine tegmentum to the VTA also activate dopamine neurons [85, 86]. Thus, our findings could be mediated by direct projections from the PLc to midbrain dopamine neurons and/or one or more indirect pathways. Whilst this was outside the translational aims of our study, further work is required to test whether pyramidal neuron activity is altered in our ketamine model and to characterise the circuit linking cortical and hippocampal PV interneurons to midbrain dopamine neurons.

Ketamine’s action on receptors other than the NMDAR, could also contribute to the observed effects [87]. Evidence suggests that ketamine’s antidepressant effects could be independent from NMDA receptors expressed in PV interneurons [87] and that deletion of dopamine D_2_ receptors from PV interneurons induces hyperlocomotion [88]. In addition, recent findings indicate that activation of dopamine D_1_ receptors on pyramidal cells in the prefrontal cortex and/or the action of a metabolite of ketamine on α-amino-3-hydroxy-5-methyl-4-isoxazolepropionic acid (AMPA) receptors might contribute to its long-lasting antidepressant effects [89, 90]. However, ketamine’s affinities at other receptors (range of Ki values = 19–131 μm) are considerably lower than its affinity for the NMDAR, and it is not clear if ketamine exhibits significant dopamine receptor occupancy in vivo at behaviourally relevant doses [91, 92]. We suggest that the effects of ketamine in our model likely involve NMDAR blockade, but a contribution from binding to other receptors cannot be excluded.

Ketamine has a short half-life (13 min) in mice [93] and brain levels of its main metabolite (2R, 6R)-hydroxynorketamine (HNK), are not detectable 4 h post administration in mice [90, 94, 95]. Our PET and RNASeq measures were acquired ~48 h following the last ketamine treatment. Thus, it is unlikely that the observed effects are a consequence of direct ketamine or HNK action. Previous studies have shown that ketamine induces a release of BDNF to increase synaptogenesis [95] and elevates MAPK signalling [96, 97]. This could present a potential mechanism by which ketamine contributes to the sustained effects observed in our model.

Interestingly, whilst CNO significantly reduced locomotor activity compared with baseline, it had no effect on dopamine synthesis capacity. It should be noted that locomotor activity was measured shortly after CNO administration whilst dopamine synthesis capacity was measured 2 days following CNO administration. Electrophysiology recordings show that dopamine neurons recover quickly upon washout of CNO from solution [40]. Thus, our findings indicate that CNO does not induce lasting changes in dopamine synthesis capacity, but is able to block the effects of ketamine on dopamine synthesis capacity.

A strength of our study is that it utilised a PET imaging approach that parallels the technique used in human studies [6, 30, 33], supporting the translational relevance of the findings. One consideration for chemogenetic approaches is the cell-type and regional specificity of expression. Injection of viral constructs in wild-type mice revealed no detectable expression (Supplementary Fig. 5), and CNO administration in transgenic mice expressing a control construct had no effect on the ketamine-induced increase in dopamine synthesis capacity and locomotor activity. Limitations include that we did not measure other aspects of dopamine function or investigate other brain regions. Moreover, we did not test whether the effects of SEP-856 are predominately mediated via TAAR1 agonism and its action on dopamine neuron firing, or also driven by the compound’s activities at other receptors [20]. To date SEP-856 has been tested for binding and/or functional activity against multiple panels of known molecular targets (ion channels, G-protein-coupled receptors and enzymes), demonstrating a range of activities at several receptors [20]. While the most notable functional activity is full agonism at TAAR1 (EC50 of 0.14 μM), SEP-856 also exhibits binding and agonist activity at the 5-HT_1A_ receptor (5-HT_1A_R), although with lower potency than for TAAR1 (EC50 of 2.3 μM) [20]. Notwithstanding this, attenuation of PCP induced hyperlocomotion by SEP-856 is partially blocked by a 5HT_1A_R antagonist [20], suggesting that its effects in our ketamine model could be partly mediated by 5HT_1A_R. Following our translational work, future pharmacology studies will help elucidate the molecular and circuit mechanisms by which SEP-856 attenuates the ketamine-induced increase in striatal dopamine synthesis capacity.

### Implications for understanding the pathophysiology of schizophrenia and the antidepressant mode of action of ketamine

PET imaging studies have repeatedly shown elevated dopamine synthesis and release capacity in schizophrenia (e.g. [7, 98] and see review [6]), and suggested that this is linked to the development of psychosis [99, 100] and changes in cortical glutamate levels [101]. Moreover, cortical and hippocampal PV interneuron density and PV protein levels have been shown to be reduced in schizophrenia (see meta-analysis [25] and [102]). Ketamine induces psychotic symptoms in healthy volunteers, and worsens symptoms in patients with schizophrenia [14, 15]. Our findings indicate that ketamine’s effect on dopamine synthesis capacity involves PV-positive interneurons in regions implicated in schizophrenia. These findings suggest that inhibition of midbrain dopamine neurons and/or activation of cortical PV interneurons could represent novel therapeutic strategies for schizophrenia. Furthermore, our finding that SEP-856, a novel TAAR1/5HT_1A_ agonist, reduces sub-chronic ketamine-induced elevation in striatal dopamine synthesis capacity provides a proof-of-concept for pharmacological attenuation of presynaptic dopamine dysfunction. The reduction in PV levels following ketamine in our model was large (Hedge’s *g* = −2.29), which compares to a moderate-large effect size (Hedge’s *g* = −0.61) reduction in PV-positive neuron immunoreactivity reported post-mortem in schizophrenia [102]. Thus, our ketamine model likely induces more marked effects on PV than are typically seen in schizophrenia.

Lastly, our data may also have implications for understanding ketamine’s antidepressant actions and its abuse potential. There is some evidence that major depression is associated with blunted dopaminergic function, including reduced levels of dopamine metabolites post-mortem and reduced dopamine synthesis capacity [103, 104]. Moreover, animal models mimicking the neurochemical changes seen in depression exhibit reduced dopamine neuron population activity [38]. Our findings that sub-chronic ketamine administration elevates striatal dopamine synthesis capacity, which persists for several days post dosing, suggest that this could contribute to ketamine’s antidepressant actions [48]. The majority of pre-clinical studies investigating the antidepressant effects of ketamine used  a 10 mg/kg dose of ketamine, but doses as high as 50 mg/kg have also been used to show antidepressant-like effects [105–110]. In human studies the optimal therapeutic dose for ketamine is debated, with 0.5 mg/kg having dissociative, psychotomimetic and antidepressant effects [111, 112], whilst 0.2 mg/kg is generally considered sub-therapeutic, although one study reported positive therapeutic effects with 0.1 mg/kg [14, 111, 113]. Thus, it would be useful to determine if lower ketamine doses than we used also result in persistent increases in dopamine synthesis capacity. It should also be noted that other mechanisms, such as augmenting ERK1/MAPK signalling and AMPA activity, are also implicated ketamine’s antidepressant actions [114]. In line with this and previous findings, we show that sub-chronic ketamine administration leads to the increase in the expression of genes involved in the pathway downstream of BDNF signalling, such as upregulation of MAPK3 suggesting this could contribute to ketamine’s antidepressant effects [96, 97].

## Conclusion

We demonstrate that sub-chronic ketamine leads to an increase in striatal dopamine synthesis capacity in the mouse, resembling the dopaminergic alteration seen in patients with schizophrenia. Our data suggest that ketamine’s effects on dopamine synthesis capacity are mediated by inhibition of PV interneurons in the cortex and vSub as well as activation of midbrain dopamine neurons, and that these alterations can be attenuated by a TAAR1 agonist with 5-HT_1A_ activity.

## Supplementary information

Supplementary Information

Supplementary Figure 1

Supplementary Figure 2

Supplementary Figure 3

Supplementary Figure 4

Supplementary Figure 5

Supplementary Figure 6

Supplementary Figure 7
